# Hemodynamic Surveillance of Unilateral Carotid Artery Stenting in Patients With or Without Contralateral Carotid Occlusion by TCD/TCCD in the Early Stage Following Procedure

**DOI:** 10.3389/fneur.2019.00958

**Published:** 2019-09-04

**Authors:** Ziguang Yan, Min Yang, Guochen Niu, Bihui Zhang, Xiaoqiang Tong, Hongjie Guo, Yinghua Zou

**Affiliations:** Department of Interventional Radiology and Vascular Surgery, Peking University First Hospital, Beijing, China

**Keywords:** carotid artery stenosis, contralateral carotid occlusion, carotid artery stenting, transcranial Doppler, transcranial color-code Doppler, cerebral hemodynamics, early stage

## Abstract

**Objective:** To evaluate the cerebral hemodynamic variations in patients with unilateral carotid artery stenosis and contralateral carotid occlusion (CCO) in hours following carotid artery stenting (CAS) by transcranial Doppler (TCD) or transcranial color-code Doppler (TCCD).

**Methods:** Sixty-five consecutive patients who underwent unilateral CAS were enrolled. Among them, 14 patients had ipsilateral severe stenosis and CCO (CCO group) while the other 51 patients had only unilateral severe carotid stenosis without CCO (UCS group). All patients underwent TCD or TCCD monitoring before, at 1 and 3 h after CAS. We monitored bilateral middle cerebral artery (MCA) peak systolic velocity (PSV), pulsatility index (PI), and blood pressure (BP), and compared that data between two groups.

**Results:** In UCS group, ipsilateral MCA PSV increased relative to baseline at 1 h (96 ± 30 vs. 85 ± 26 cm/s, 15%, *P* < 0.001) and 3 h (97 ± 29 vs. 85 ± 26 cm/s, 17%, *P* < 0.001) following CAS. Significant PI increases were observed at 1 and 3 h following CAS on the ipsilateral side. In CCO group, ipsilateral MCA PSV increased relative to baseline at 1 h (111 ± 30 vs. 83 ± 26 cm/s, 35%, *P* < 0.001) and 3 h (107 ± 28 vs. 83 ± 26 cm/s, 32%, *P* <0.001) following CAS. The magnitude of ipsilateral MCA PSV increase was significantly higher in CCO group compared with UCS group at 1 h (*P* = 0.002) and 3 h (*P* = 0.024) following CAS, while BP similarly decreased between the two groups. On the contralateral side, significant MCA PSV increases were observed following CAS in CCO group but not in UCS group. Bilateral MCA PSV increases were higher in patients with a stenosis degree of ≥90% than in patients with stenosis degree of 70–89% only in CCO group.

**Conclusion:** The ipsilateral MCA PSV and PI increase moderately in the initial hours after unilateral CAS in patients without CCO. In patients with CCO, the ipsilateral, and contralateral MCA PSV increase significantly in the early stage following CAS. CCO is a factor of the increased blood flow velocity in ipsilateral MCA after unilateral CAS.

## Introduction

Contralateral carotid artery occlusion (CCO) was found in 5–15% of carotid artery stenosis (CS) patients (1–4). According to the North American Symptomatic Carotid Endarterectomy Trial (NASCET), CCO has been demonstrated as an independent risk factor for carotid endarterectomy CEA (1, 2, 5, 6). While, carotid artery stenting (CAS) is suggested as an alternative for the treatment of patients with CS and CCO (2, 3). A recent meta-analysis about cerebral hyperperfusion syndrome (CHS) encouraged further investigation on cerebral hemodynamic monitoring (7). Besides, a crucial risk factor of periprocedural stroke following CAS is hemodynamic disturbance (HD), which often occurs within 6 h after CAS (8–11). However, only a few studies have evaluated cerebral hemodynamic changes in the early stage following CAS in patients with CCO. Transcranial Doppler (TCD) and transcranial color-code Doppler (TCCD) are bedside examinations and can be used for routine clinical monitoring of cerebral hemodynamic changes immediately after CAS (12). Our study used TCD and TCCD to assess the immediate effect on cerebral hemodynamics after CAS in patients with and without CCO.

## Materials and Methods

### Subjects

All patients who underwent CAS in Department of Interventional Radiology and Vascular Surgery at Peking University First Hospital from Jan, 2013 to Dec, 2018 were enrolled in this study. One hundred forty-eight patients underwent CAS, of whom 27 patients had no bone window. TCD were performed in 121 patients and 56 of them were excluded because of simultaneous bilateral carotid stenting (nine patients), simultaneous vertebral or subclavian artery stenting (16 patients), carotid artery near occlusion (20 patients), or moderate-severe contralateral carotid artery stenosis (11 patients). Carotid stenosis was diagnosed using ultrasound and computed tomography angiography (CTA), and finally Four-vessel angiography. Among all the remaining 65 patients, 14 patients were diagnosed severe CS with CCO, 51 patients had severe unilateral CS.

### CAS Protocol

**Figure 1:**
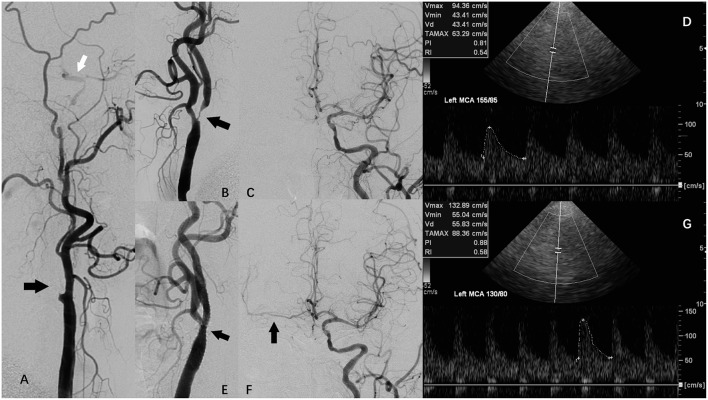
A patient with carotid artery stenosis and contralateral carotid occlusion (CCO) underwent carotid artery stenosis (CAS), and periprocedural transcranial color-code Doppler (TCCD) monitoring. **(A)** Digital subtraction angiography (DSA) showing right internal carotid artery (ICA) occlusion (black arrow) and right external carotid artery supplying right middle cerebral artery (MCA) via collateral circulation of ophthalmic artery. **(B)** DSA showing left ICA severe stenosis (arrow). **(C)** DSA showing left ICA supplying right anterior cerebral artery (ACA) via anterior communicating artery. **(D)** TCCD before CAS showing left MCA peak systolic velocity (PSV) was 94 cm/s, while systolic blood pressure (SBP) was 155 mmHg. **(E)** DSA showing left ICA following CAS. **(F)** DSA showing left ICA supplying right ACA and MCA via anterior communicating artery following CAS. **(G)** TCCD at 1 h after CAS maintained a constant depth, angle of insonation, and an original probe-skin contact point, showing left MCA PSV was 133 cm/s, while SBP was 130 mmHg.

CAS was performed in symptomatic (at least 2 weeks after onset of symptom) or asymptomatic patients with >70% stenosis (NASCET criteria). Written informed consent was obtained from all of the patients that underwent CAS. At least 72 h before the procedure, all patients received antithrombotic premedication (100 mg aspirin and 75 mg clopidogrel). Transbrachial approach was used in one patient because of aortic-iliac artery occlusion. Transfemoral approach with local anesthesia using 2% lidocaine was used in all the other cases. Distal embolic protection device was used in all the patients. We routinely applied pre-dilation with a 4.0–5.0 mm balloon catheter (Boston Scientific, Natick, MA), and selected the appropriate stent device (Precise RX, Cordis Endovascular; Acculink, Abbott Vascular; and Carotid Wallstent, Boston Scientific) according to the anatomic location and the diameter of the artery at the operater's discretion. We would not perform post-dilation unless the residual stenosis was more than 30%. The completion angiogram of carotid artery and distal cerebral vasculature was performed after stent deployment (Figure 1).

### Transcranial Doppler

Examination was performed using a 2-MHz probe connected to a TCD machine (TC2021, EME, Companion III, Germany) or a transcranial color-code Doppler (TCCD) machine (GE LOGIOe) fitted with 2.0-MHz sector array transducer. The ipsilateral and/or contralateral middle cerebral artery (MCA) was insonated through the temporal window at a depth of 46–60 mm. We recorded peak systolic velocity (PSV) and pulsatility index (PI) at baseline on the day before CAS, and again at about 1 and 3 h following the CAS procedure. To maintain a constant depth, angle of insonation, and an original probe-skin contact point (Figure 1), all TCD or TCCD examinations in the patients were performed by an identical physician. Post-CAS hyperperfusion was defined as the MCA-PSV exceeded 2-fold of the pre-CAS TCD measurement (13, 14).

### Blood Pressure Control

Blood pressure (BP) was monitored and controlled throughout the periprocedure period. Before balloon predilation, systolic BP was controlled below 160 mmHg. After predilation and stent deployment, systolic blood pressure was preliminarily controlled between 90 and 140 mmHg for unilateral CAS patients. If potential hyperperfusion or hypoperfusion were detected by the first TCD, BP would be further adjusted. Hemodynamic depression (HD) was defined as periprocedural hypotension (BP <90/60 mmHg) or bradycardia (heart rate <50 beats/min). Persistant HD was defined as HD persisted for at least 1 h. Dopamine or/and atropine were used for HD patients. Urapidil or/and nicardipine were administered intravenously to lower BP, which was measured during the examination using a standard BP cuff.

### Statistical Analysis

We performed all statistical analyses using IBM SPSS software (version 23.0). TCD data are presented as mean ± standard deviation (SD). PSV and PI values the day before CAS, and at both 1 and 3 h following CAS were evaluated using paired *t*-test, after repeated measure ANOVA. Bonferroni correction was used, and statistical significance was considered to be *P* < 0.05/3 (= 0.0167). Variations between groups were compared using independent *t*-test and *P* < 0.05 was considered statistically significant.

### Study Approval

The protocol for this study was approved by the institutional review board at the Peking University First Hospital in accordance with the Chinese clinical research ethics guidelines. All data were obtained from the Peking University First Hospital, Department of Interventional Radiology and Vascular Surgery, after anonymization.

## Results

All CAS procedures were successful and without adverse events. Among the 65 patients enrolled, 14 patients had ipsilateral severe stenosis and CCO (CCO group), the other 51 patients had only unilateral severe carotid stenosis without CCO (UCS group). The mean (±SD) age of UCS group was 66 ± 8 years. Of these patients, 24 (47%) were symptomatic, while the remaining 27 patients (53%) were asymptomatic. Forty-two patients (82%) of UCS group were male. The average degree of ICA stenosis of UCS group was 82 ± 8%. The mean (±SD) age of CCO group was 67 ± 7 years. Of these patients, 10 (71%) were symptomatic, while the remaining four patients (29%) were asymptomatic. Twelve patients (86%) of CCO group were male. The average degree of ICA stenosis of CCO group was 81 ± 11%. Angiography showed opened anterior communicating branch in all the CCO patients. Contralateral MCA was supplied by anterior communicating branch in four patients before CAS and in six patients after CAS.

The demographic data are shown in Table 1. Three different types of stent were used in both groups. There were no instances of severe hyperperfusion syndrome, renal failure, deaths or disabling strokes in any of the participants in the month following CAS. Three patients in UCS group had minor stroke in the early phase following CAS. Four patients in UCS group and two patients in CCO group had persistent HD, which we treated with dopamine during the 24-h period following CAS (Table 1). In both groups, the mean BP decreased after CAS. The mean BP values did not significantly differ between the two groups, either at baseline or post-CAS.

**Table 1:** Patient demographic, stents and outcome data.

**Variable**	**UCS group (*n* = 51)**	**CCO group (*n* = 14)**	***P***
Male	42 (82%)	12 (86%)	0.562
Age≥ 70 years	19 (37%)	5 (36%)	0.916
Hypertension	41 (80%)	9 (64%)	0.205
Diabetes mellitus	23 (45%)	3 (21%)	0.096
Smoke	32 (63%)	10 (71%)	0.394
Asymptomatic	27 (53%)	4 (29%)	0.093
Hyperlipidemia	42 (82%)	11 (79%)	0.711
Different stent:			
Precise RX	36 (70%)	11 (79%)	0.411
Wallstent	6 (12%)	1 (7%)	0.528
Acculink	9 (18%)	3 (14%)	0.506
Outcome:			
Stenosis degree ≥ 90%	19 (37%)	5 (36%)	0.916
Pre-CAS iMCA PSV (cm/s)	85 ± 26	83 ± 26	0.829
Transient or Permanent HD	16 (31%)	5 (36%)	0.497
Transient HD	12 (24%)	3 (21%)	0.590
Persistent HD	4 (8%)	2 (14%)	0.384
Minor stroke	3 (6%)	0	0.477
Hyperperfusion syndrome	0	0	
Myocardial infarction, renal failure, or other events	0	0	

*NO, near occlusion; ICA, internal carotid artery; iMCA, ipsilateral middle cerebral artery; PSV, peak systolic velocity; CAS, carotid artery stenting; HD, hemodynamic depression*.

*P < 0.05 was considered statistically significant*.

TCD examinations were performed in all the 65 patients before CAS, and at 1 and 3 h after CAS. Among them, three patients in UCS group and two patients in CCO group received only ipsilateral TCD examination because of unilateral absence of bone window, or contralateral MCA occlusion. In UCS group, at 1 h after CAS, TCD showed a significant PSV increase in the ipsilateral MCA (from 85 ± 26 to 96 ± 30 cm/s, 15%, *P* < 0.001). The average PI also increased in the ipsilateral MCA (from 0.85 ± 0.16 to 0.94 ± 0.24, *P* = 0.003). At 3 h after CAS, the PSV in the ipsilateral MCA was also significantly increased compared to the value before CAS (from 85 ± 26 to 97 ± 29 cm/s, 17%, *P* <*0.0*01), but similar to the value 1 h after CAS (*P* = 0.514). A significant PI increase was observed 3 h after CAS (from 0.85 ± 0.16 to 1.0 ± 0.25, *P* < 0.001). On the contralateral side, there was no significant PSV or PI increase in the MCA for either 1 or 3 h after CAS (Table 2).

**Table 2:** Parameters of hemodynamic changes in UCS group.

	**Pre-CAS**	**1 h post-CAS**	***P***	**3 h post-CAS**	***P***
BP (mm Hg)	143 ± 16	116 ± 12	<0.001	117 ± 12	<0.001
iMCA PSV (cm/s)	85 ± 26	96 ± 30	<0.001	97 ± 29	<0.001
iMCA PI	0.85 ± 0.16	0.94 ± 0.24	0.003	1.0 ± 0.25	<0.001
cMCA PSV (cm/s)	89 ± 24	90 ± 27	0.631	90 ± 26	0.395
cMCA PI	0.93 ± 0.15	0.93 ± 0.21	0.953	0.95 ± 0.19	0.234

*CAS, carotid artery stenting; BP, blood pressure; iMCA, ipsilateral middle cerebral artery; PSV, peak systolic velocity; PI, pulsatility index; cMCA, contralateral middle cerebral artery*.

*P < 0.017 (after Bonferroni correction) was considered statistically significant*.

In CCO group, at 1 h after CAS, TCD showed a significant PSV increase in the ipsilateral MCA (from 83 ± 26 to 111 ± 30 cm/s, 35%, *P* < 0.001). At 3 h after CAS, the PSV value in the ipsilateral MCA was also significantly increased compared to prior CAS (from 83 ± 26 to 107 ± 28 cm/s, 32%, *P* < 0.001), but similar to the value at 1 h after CAS (*P* = 0.144). There was no significant PI increase in the ipsilateral MCA for either 1 or 3 h after CAS. On the contralateral side, the MCA PSV increased in 1 h after CAS (69 ± 16 vs. 90 ± 29, 28%, *P* = 0.001) and 3 h after CAS (69 ± 16 vs. 86 ± 29, 22%, *P* = 0.005) compared with the value before CAS. There was no significant PI increase in the contralateral MCA for either 1 or 3 h after CAS (Table 3).

**Table 3:** Parameters of hemodynamic changes in CCO group.

	**Pre-CAS**	**1 h post-CAS**	***P***	**3 h post-CAS**	***P***
BP (mm Hg)	148 ± 12	125 ± 17	<0.001	122 ± 19	<0.001
iMCA PSV (cm/s)	83 ± 26	111 ± 30	<0.001	107 ± 28	<0.001
iMCA PI	0.85 ± 0.16	0.90 ± 0.17	0.191	0.92 ± 0.19	<0.097
cMCA PSV (cm/s)	69 ± 16	90 ± 29	0.001	86 ± 29	0.005
cMCA PI	0.74 ± 0.13	0.77 ± 0.15	0.231	0.75 ± 0.13	0.634

*CAS, carotid artery stenting; BP, blood pressure; iMCA, ipsilateral middle cerebral artery; PSV, peak systolic velocity; PI, pulsatility index; cMCA, contralateral middle cerebral artery*.

*P < 0.017 (after Bonferroni correction) was considered statistically significant*.

The increase rate of BP had no significant difference at 1 or 3 h after CAS between the two groups. There was no significant difference of the average pre-CAS ipsilateral MCA PSV between the two groups (*P* = 0. 829). The magnitude of ipsilateral MCA PSV increases in CCO group significantly exceeded that observed in UCS group at both 1 h after CAS (35 vs. 15%, *P* = 0.002), and 3 h after CAS (32 vs. 17%, *P* = 0.024; Table 4). In CCO group, five patients had a ≥90% stenosis degree. In these patients, the magnitude of ipsilateral MCA PSV increase was 53 ± 17% at 1 h and 52 ± 21% at 3 h after CAS, significantly higher than the magnitude of 26 ± 11% (*P* = 0.004) at 1 h and 21 ± 19% (*P* = 0.018) at 3 h in the other nine patients. In UCS group, at 1 or 3 h after CAS, the magnitude of ipsilateral MCA PSV increase had no statistically significant difference whether stenosis degree was ≥90% (Table 4). In both groups, the magnitude of ipsilateral MCA PSV increase had no significant difference with the varied type of Willis circle, whether the patients were ≥70 years old or whether the patients were asymptomatic (data not shown).

**Table 4:** Increase rate of ipsilateral MCA PSV following CAS in UCS group and CCO group.

	**1 h post-CAS**		**3 h post-CAS**	
**Average increase rate**	**BP**	**iMCA PSV**	**BP**	**iMCA PSV**
UCS group (*n* = 51)	−18%	15%	−18%	17%
CCO group (*n* = 14)	−15%	35%	−17%	32%
*P*	0.331	0.002	0.930	0.024
CCO group ≥ 90% (*n* = 5)	−17%	53%	−17%	52%
CCO group <90% (*n* = 9)	−14%	26%	−18%	21%
*P*	0.656	0.004	0.927	0.018
UCS group ≥ 90% (*n* = 19)	−18%	22%	−17%	24%
UCS group <90% (*n* = 32)	−19%	11%	−18%	12%
*P*	0.715	0.089	0.753	0.056

*CAS, carotid artery stenting; BP, blood pressure; iMCA, ipsilateral middle cerebral artery; PSV, peak systolic velocity; PI, pulsatility index*.

*P < 0.05 was considered statistically significant*.

## Discussion

Patients with CS and CCO carry a higher incidence of complication following CEA and CAS (15). A previous meta-analysis recommended CAS, rather than CEA in patients with CCO (2). HD and CHS are two different complications of CAS related to cerebral hemodynamic changes, both may occur within 6 h following CAS (16–19). However, only few studies have focused on cerebral hemodynamic changes in the early stage following CAS, especially in the patients with CCO. The present research clarified the changes of bilateral MCA PSV in the early stage after unilateral CAS in patients with or without CCO.

A previous research demonstrated an about 20% increase of the ipsilateral MCA PSV in the early stage following CAS (12). However, sample in that research had some extent heterogeneity. The present research excluded several potential risk factors, such as simultaneous bilateral carotid stenting, simultaneous vertebral or subclavian artery stenting, carotid artery near occlusion, or contralateral carotid artery stenosis (12, 20). Therefore, the variation of cerebral blood flow velocity after CAS in patients with simple unilateral carotid artery stenosis could be observed for the first time. Meanwhile, the influence of CCO on MCA PSV change after unilateral CAS could be demonstrated more clearly. Concerning the changes of PSV, the previous research stated that there were no significant differences between patients with ≥90% stenosis and those with 70–89% stenosis. The present research shows that although in UCS group the increment in ipsilateral MCA PSV in patients with ≥90% stenosis is greater, there is still no statistical significance. In CCO group, however, it is observed that ipsilateral MCA PSV increased significantly higher in patients with a ≥90% stenosis, which might be attributed to the impaired cerebral hemodynamic autoregulation.

Following CAS, there is a 3.1–6.8% risk of CHS, that most likely occurs in the early post-procedural period (7). Abou-Chebl et al. (11) has suggested that patients with severe bilateral carotid stenosis were predisposed to CHS, and patients with CCO should require more intensive hemodynamic monitoring after CAS. However, in the present study, no patient had more than 100% increase of the MCA PSV following the procedure and none CHS occurred. The increase of ipsilateral MCA PSV was at an average of 35 and 32% at 1 and 3 h following CAS, respectively. The maximum magnitude of MCA PSV increase was 84% in the ipsilateral side and 67% in the contralateral side. These results suggest that for patients with CCO, under a strict BP control and cerebral hemodynamic monitoring after CAS, the risk for CHS can be reduced.

Regional cerebral blood flow is proportional to blood flow velocity in the MCA (21, 22). A previous research measured cerebral blood flow by SPECT within 2 h following CAS in patients with CCO (23). In that research, no significant difference was found in resting cerebral blood flow in both hemispheres immediately after CAS, which differed from the present research. Besides, the previous research did not include comparisons with a control group. To our knowledge, there are no other study focus on the immediate cerebral hemodynamic changes in CCO patients following CAS.

In the control group, there were only a little bit more than 15% average increase of ipsilateral MCA PSV at 1 and 3 h following procedure, perhaps due to a relatively normal cerebral autoregulation (24). In this article, we analyzed not only PSV but PI. Increase of PI indicates that the waveform becomes steeper. The PI is not dependent solely on cerebrovascular resistance but a product of the interplay between cerebral perfusion pressure, pulse amplitude of arterial pressure, cerebrovascular resistance and compliance of the cerebral arterial bed as well as the heart rate (25). Notably, PI increased significantly in the ipsilateral MCA following CAS in UCS group. This finding reveals that vasoconstriction of resistance arterioles can accommodate the substantially increased MCA blood flow that follows CAS (18, 25, 26). It is probably because CCO could reduce the cerebral vascular reactivity and the cerebral perfusion reserve (27–29), no PI changes were found in CCO group. Hence the increases of bilateral MCA PSV as well as the cerebral blood was greater than that of patients without CCO.

The present study did not include some parameters such as intracranial pressure or cerebrovascular reactivity. Only to measure the MCA velocities can facilitate the TCD examination and ensure the data of all the patients could be collected on time. Some medications, such as statins, vasopressor or antihypertensives, may have an impact on cerebral circulation (30). The potential confounding role of these medications will be studied in future researches. There were two limitations in the present research. First was the limited sample size. The present research observed greater increases of ipsilateral MCA PSV in patients with an original stenosis degree of ≥90%. However, it needs further confirmation by future large sample study. The second limitation was the gender imbalance. This was because TCD or TCCD were not feasible in patients with a poor temporal window, and female accounted for a high incidence.

## Conclusions

In patients with unilateral severe carotid stenosis and without CCO, the ipsilateral MCA PSV and PI increase moderately in the initial hours after unilateral CAS. In patients with CCO, the ipsilateral and contralateral MCA PSV significantly increase in the early stage following CAS. The MCA PSV of both sides may increase more in CCO patients with an original stenosis degree of ≥90%. CCO is a factor of the increased blood flow velocity in ipsilateral MCA after unilateral CAS.

## Data Availability

The datasets generated for this study are available on request to the corresponding author.

## Ethics Statement

The protocol for this study was approved by the institutional review board at the Peking University First Hospital in accordance with the Chinese clinical research ethics guidelines. All data were obtained from the Peking University First Hospital, Department of Interventional Radiology and Vascular Surgery, after anonymization. Informed consent was obtained from all of the patients that underwent CAS.

## Author Contributions

ZY and MY: conception or design of the work, drafting and critical revision of the article, and final approval of the version to be published. GN, BZ, XT, and HG: data collection, data analysis, and interpretation. YZ: conception or design of the work.

### Conflict of Interest Statement

The authors declare that the research was conducted in the absence of any commercial or financial relationships that could be construed as a potential conflict of interest.

## References

[1] Maatz W Köhler J Botsios S John V Walterbusch G. Risk of stroke for carotid endarterectomy patients with contralateral carotid occlusion. Ann Vasc Surg. (2008) 22:45–51. 10.1016/j.avsg.2007.07.034 18083336

[2] Faggioli G Pini R Mauro R Freyrie A Gargiulo M Stella A. Contralateral carotid occlusion in endovascular and surgical carotid revascularization: a single centre experience with literature review and meta-analysis. Eur J Vasc Endovasc Surg. (2013) 46:10–20. 10.1016/j.ejvs.2013.03.021 23639235

[3] Mehta RH Zahn R Hochadel M Mudra H Ischinger T Hauptmann KE. Effectiveness and safety of carotid artery stenting for significant carotid stenosis in patients with contralateral occlusion (from the German ALKK-CAS Registry experience). Am J Cardiol. (2009) 104:725–31. 10.1016/j.amjcard.2009.04.038 19699352

[4] International Carotid Stenting Study investigators Ederle J Dobson J Featherstone RL Bonati LH van der Worp HB. Carotid artery stenting compared with endarterectomy in patients with symptomatic carotid stenosis (International Carotid Stenting Study): an interim analysis of a randomised controlled trial. Lancet. (2010) 375:985–97. 10.1016/S0140-6736(10)60239-5 20189239 PMC2849002

[5] North American Symptomatic Carotid Endarterectomy Trial Methods, patient characteristics, and progress. Stroke. (1991) 22:711–20. 10.1161/01.STR.22.6.711 2057968

[6] Executive Committee for the Asymptomatic Carotid Atherosclerosis Study Endarterectomy for asymptomatic carotid artery stenosis. JAMA. (1995) 273:1421–8. 10.1001/jama.273.18.1421 7723155

[7] Huibers AE Westerink J de Vries EE Hoskam A den Ruijter HM Moll FL. Cerebral hyperperfusion syndrome after carotid artery stenting: a systematic review and meta-analysis. Eur J Vasc Endovasc Surg. (2018) 56:322–33. 10.1016/j.ejvs.2018.05.012 30196814

[8] Huibers A Calvet D Kennedy F Czuriga-Kovács KR Featherstone RL Moll FL Mechanism of procedural stroke following carotid endarterectomy or carotid artery stenting within the International Carotid Stenting Study (ICSS) randomised trial. Eur J Vasc Endovasc Surg. (2015) 50:281–8. 10.1016/j.ejvs.2015.05.017 26160210 PMC4580136

[9] Gupta R Abou-Chebl A Bajzer CT Schumacher HC Yadav JS. Rate, predictors, and consequences of hemodynamic depression after carotid artery stenting. J Am Coll Cardiol. (2006) 47:1538–43. 10.1016/j.jacc.2005.08.079 16630988

[10] Widecka-Ostrowska K Modrzejewski A Goracy J. Haemodynamic depression during carotid angioplasty and stenting. Pol J Radiol. (2010) 75:34–7. 22802802 PMC3389897

[11] Abou-Chebl A Yadav JS Reginelli JP Bajzer C Bhatt D Krieger DW. Intracranial hemorrhage and hyperperfusion syndrome following carotid artery stenting: risk factors, prevention, and treatment. J Am Coll Cardiol. (2004) 43:1596–601. 10.1016/j.jacc.2003.12.039 15120817

[12] Yan Z Yang M Niu G Zou Y. Analysis of hemodynamic changes in early stage after carotid stenting by transcranial Doppler-A Preliminary Study. Ann Vasc Surg. (2017) 45:85–91. 10.1016/j.avsg.2017.06.124 28687500

[13] Pennekamp CW Moll FL De Borst GJ. Role of transcranial Doppler in cerebral hyperperfusion syndrome. J Cardiovasc Surg. (2012) 53:765–71. 23207559

[14] Van Mook WN Rennenberg RJ Schurink GW van Oostenbrugge RJ Mess WH Hofman PA. Cerebral hyperperfusion syndrome. Lancet Neurol. (2005) 4:877–88. 10.1016/S1474-4422(05)70251-9 16297845

[15] Nejim B Dakour Aridi H Locham S Arhuidese I Hicks C Malas MB Carotid artery revascularization in patients with contralateral carotid artery occlusion: stent or endarterectomy? J Vasc Surg. (2017) 66:1735–48.e1. 10.1016/j.jvs.2017.04.055 28666824

[16] Kablak-Ziembicka A Przewlocki T Pieniazek P Musialek P Tekieli L Rosławiecka A. Predictors of cerebral reperfusion injury after carotid stenting: the role of transcranial color-coded Doppler ultrasonography. J Endovasc Ther. (2010) 17:556–63. 10.1583/09-2980.1 20681776

[17] Coutts SB Hill MD Hu WY. Hyperperfusion syndrome: toward a stricter definition. Neurosurgery. (2003) 53:1053–58. 10.1227/01.NEU.0000088738.80838.74 14580271

[18] Sanchez-Arjona MB Sanz-Fernandez G Franco-Macias E Gil-Peralta A. Cerebral hemodynamic changes after carotid angioplasty and stenting. Am J Neuroradiol. (2007) 28:640–4. 17416813 PMC7977359

[19] Lavoie P Rutledge J Dawoud MA Mazumdar M Riina H Gobin YP. Predictors and timing of hypotension and bradycardia after carotid artery stenting. Am J Neuroradiol. (2008) 29:1942–7. 10.3174/ajnr.A1258 18719034 PMC8118931

[20] Yan Z Yang M Niu G Tong X Zou Y. Cerebral Hemodynamic Variations in the early stage after carotid artery stenting in patients with and without near occlusion. Ann Vasc Surg. (2019) 59:5–11. 10.1016/j.avsg.2019.01.020 31009728

[21] Sorteberg W Lindegaard KF Rootwelt K Dahl A Russell D Nyberg-Hansen R. Blood velocity and regional blood flow in defined cerebral artery systems. Acta Neurochir. (1989) 97:47–52. 10.1007/BF01577739 2785744

[22] Muller M Voges M Piepgras U Schimrigk K. Assessment of cerebral vasomotor reactivity by transcranial Doppler utrasound and breath-holding. A comparison with acetazolamide as vasodilatory stimulus. Stroke. (1995) 26:96–100. 10.1161/01.STR.26.1.96 7839406

[23] Oka F Ishihara H Kato S Higashi M Suzuki M. Cerebral hemodynamic benefits after contralateral carotid artery stenting in patients with internal carotid artery occlusion. Am J Neuroradiol. (2013) 34:616–21. 10.3174/ajnr.A3250 22918426 PMC7964908

[24] Diehl RR. Cerebral autoregulation studies in clinical practice. Eur J Ultrasound. (2002) 16:31–6. 10.1016/S0929-8266(02)00048-4 12470848

[25] De Riva N Budohoski KP Smielewski P Kasprowicz M Zweifel C Steiner LA. Transcranial Doppler pulsatility index: what it is and what it isn't. Neurocrit Care. (2012) 17:58–66. 10.1007/s12028-012-9672-6 22311229

[26] Nowacki P Zywica A Podbielski J Kornacewicz-Jach Z Drechsler H Drechsler D. Middle cerebral artery flow after angioplasty and stenting of symptomatic internal carotid artery stenosis. Neurol Neurochir Pol. (2009) 43:9–15. 19353439

[27] Ogasawara K Ogawa A Yoshimoto T. Cerebrovascular reactivity to acetazolamide and outcome in patients with symptomatic internal carotid or middle cerebral artery occlusion: a xenon-133 single- photon emission computed tomography study. Stroke. (2002) 33:1857–62. 10.1161/01.STR.0000019511.81583.A8 12105366

[28] Kuroda S Kamiyama H Abe H Houkin K Isobe M Mitsumori K. Acetazolamide test in detecting reduced cerebral perfusion reserve and predicting long-term prognosis in patients with internal carotid artery occlusion. Neurosurgery. (1993) 32:912–18. 10.1097/00006123-199306000-00005 8327091

[29] Maltezos CK Papanas N Papas TT Georgiadis GS Dragoumanis CK Marakis J. Changes in blood flow of anterior and middle cerebral arteries following carotid endarterectomy: a transcranial Doppler study. Vasc Endovascular Surg. (2007) 41:389–96. 10.1177/1538574407302850 17942853

[30] Giannopoulos S Katsanos AH Tsivgoulis G Marshall RS. Statins and cerebral hemodynamics. J Cereb Blood Flow Metab. (2012) 32:1973–6. 10.1038/jcbfm.2012.122 22929438 PMC3494001

